# Deep learning segmentation of hyperautofluorescent fleck lesions in Stargardt disease

**DOI:** 10.1038/s41598-020-73339-y

**Published:** 2020-10-05

**Authors:** Jason Charng, Di Xiao, Maryam Mehdizadeh, Mary S. Attia, Sukanya Arunachalam, Tina M. Lamey, Jennifer A. Thompson, Terri L. McLaren, John N. De Roach, David A. Mackey, Shaun Frost, Fred K. Chen

**Affiliations:** 1 Centre for Ophthalmology and Visual Science (Incorporating Lions Eye Institute), The University of Western Australia, 2 Verdun Street, Nedlands, WA Australia; 2 The Australian E-Health Research Centre, Health and Biosecurity, CSIRO, Brisbane, QLD Australia; 3 Australian Inherited Retinal Disease Registry and DNA Bank, Department of Medical Technology and Physics, Sir Charles Gairdner Hospital, Nedlands, WA Australia; 4 Department of Ophthalmology, Sir Charles Gairdner Hospital, Nedlands, WA Australia; 5 Department of Ophthalmology, Royal Perth Hospital, Perth, WA Australia; 6 Department of Ophthalmology, Perth Children’s Hospital, Perth, WA Australia

**Keywords:** Biomarkers, Medical research

## Abstract

Stargardt disease is one of the most common forms of inherited retinal disease and leads to permanent vision loss. A diagnostic feature of the disease is retinal flecks, which appear hyperautofluorescent in fundus autofluorescence (FAF) imaging. The size and number of these flecks increase with disease progression. Manual segmentation of flecks allows monitoring of disease, but is time-consuming. Herein, we have developed and validated a deep learning approach for segmenting these Stargardt flecks (1750 training and 100 validation FAF patches from 37 eyes with Stargardt disease). Testing was done in 10 separate Stargardt FAF images and we observed a good overall agreement between manual and deep learning in both fleck count and fleck area. Longitudinal data were available in both eyes from 6 patients (average total follow-up time 4.2 years), with both manual and deep learning segmentation performed on all (n = 82) images. Both methods detected a similar upward trend in fleck number and area over time. In conclusion, we demonstrated the feasibility of utilizing deep learning to segment and quantify FAF lesions, laying the foundation for future studies using fleck parameters as a trial endpoint.

## Introduction

Stargardt disease (STGD1, OMIM #248200) refers to an inherited retinal disorder which causes progressive vision loss. The hallmark of STGD1 is the accumulation of bisretinoid fluorophores in the photoreceptor outer segments and lipofuscin-like fluorophores in the retinal pigment epithelium (RPE) due to the impaired flippase function of a retina-specific transmembrane protein, the adenosine triphosphate-binding cassette subfamily A member 4 (ABCA4)^1^. Several investigational drugs have been developed to inhibit or block biochemical pathways that lead to the formation of the bisretinoid fluorophores and their conversion to the toxic lipofuscin-like fluorophores in the RPE^2–6^. Some clinical trials have utilized fundus autofluorescence (FAF) imaging to measure fluorophore concentration and distribution in the retina as an endpoint to assess disease progression rate and therapeutic efficacy of these new agents^7–9^.

The earliest stage of STGD1 is characterized by an increase in the background FAF signal due to widespread accumulation of lipofuscin-like fluorophores within the RPE layer^10^. A recent multimodal imaging study showed that the formation of discrete fleck lesions, characterized by yellow pisciform deposits, which spread centrifugally and circumferentially around the fovea, correspond to clusters of degenerating photoreceptor cells^8^. These intensely hyperautofluorescent flecks, with FAF signal arising from photoreceptor bisretinoid fluorophores, make them a particularly prominent features on FAF imaging, even before they are clearly visible on clinical fundoscopy^11,12^. Over a period of years, these hyperautofluorescent flecks evolve through their own natural life cycle of enlargement, confluence and then regression into dark regions on FAF imaging due to death of the RPE^9^. Currently, FAF is used as a primary endpoint for novel treatment in STGD1 clinical trials (ClinicalTrials.gov identifiers NCT01736592, NCT03772665). The key parameter in FAF analysis is the quantification of the area where the autofluorescence signal is lacking due to the death of RPE cells^7,9^. However, a proportion of patients with childhood or late-onset diseases cannot be monitored in this way as they present prior to RPE loss. At this pre-atrophic stage, FAF has been used to quantify the signal intensity of the background diffuse autofluorescence (also known as quantitative autofluorescence, qAF, available on Heidelberg Spectralis, Heidelberg Engineering, Heidelberg, Germany) as a biomarker of RPE lipofuscin-like fluorophore accumulation^13^. However, qAF is not widely available and is no longer compatible with the most recent upgrade of the Heidelberg device software. Furthermore, patients are generally not diagnosed until flecks are visible and by this stage, quantification of AF can be confounded by the number and size of these flecks as they are highly hyperautofluorescent. Hence, tracking the evolution of fleck lesions using FAF individually or collectively over time as an alternative approach to monitoring STGD1 disease progression warrants further investigation. Automated segmentation of the central atrophic region on FAF imaging has been described in retinitis pigmentosa^14^. To the best of our knowledge, there has only been one study that described a thresholding algorithm in detecting flecks in STGD1 fundus images^15^. However, this algorithm was developed to identify but not quantify retinal flecks.

Herein, we used a set of manually segmented FAF images (ground truth, current gold standard) from patients with genetically confirmed STGD1 to develop a deep learning model for automated segmentation of flecks. We then compared the performance of the deep learning algorithm to manual segmentation by analyzing the same sets of longitudinal images using both approaches.

## Results

### Patient demographics

Forty-seven FAF images from 24 subjects (12 males, 12 females) from 19 families with a molecular diagnosis of STGD1 were utilized in the study (Table 1). The mean (range) age at which the first FAF images were obtained was 48 (12–89) years. Two patients (Patients 15 and 19) were of Asian descent while the remaining twenty-two were Caucasian. The median (range) age of onset of symptoms (or first sign of retinal lesion) was 33 (9–89) years. Seven (29%) patients had Fishman grade of 1–2 (i.e. no macular atrophy) and the remaining 17 (71%) had Fishman grade of 3 (8 with flecks localized to the posterior pole and 9 with flecks extending beyond the posterior pole). None had Fishman grade of 4.

**Table 1:** Summary of patient demographics, clinical data and genetic diagnoses.

Pid^a^	Sex	Age onset	Age imaging	RE BCVA^b^	LE BCVA^b^	RE clinical grading^c^	LE clinical grading^c^	Paternal allele	Maternal allele
1^d^	M	55	60	20/16	20/20	2	2	c.4253+43G>A;5836-145C>A	c.5177C>A;5603A>T
2	F	12	15	20/200	20/160	3C	3C	c.5461-10T>C	c.4139C>T
3a^g^	F	89	89	20/32	20/25	1	1	c.6498C>T^e^	c.2564G>A^e^
3b^g^	M	58	58	20/32	20/20	3B	3B	c.3113C>T^f^	c.2564G>A
4a	F	22	26	20/25	20/20	2	2	c.5691G>T	c.768G>T
4b	F	18	24	20/40	20/50	2	2	c.5691G>T	c.768G>T
5	F	72	70	20/25	20/25	2	2	c.4222T>C; 4918C>T^e^	c.5603A>T^e^
6	F	70	76	20/25	20/20	3C	3C	c.2576A>G^e^	c.2041C>T^e^
7a	M	17	27	20/1200	20/1200	3C	3C	c.6079C>T^f^	c.768G>T
7b	M	16	29	20/160	20/160	3C	3C	c.6079C>T^f^	c.768G>T
8a	M	9	13	20/320	20/320	3C	3C	c.5461-10T>C	c.4320delT
8b	M	11	12	20/40	20/32	2	2	c.5461-10T>C	c.4320delT
9a	F	36	57	20/20	20/20	3C	3C	c.6079C>T	c.4577C>T
9b	M	51	56	20/1000	20/250	3C	3C	c.6079C>T	c.4577C>T
10	M	30	44	20/16	20/16	3B	3B	c.3608G>A; 4537dupC^f^	c.3113C>T
11	F	50	55	20/160	20/32	3C	3C	c.2827C>T; c.5603A>T	c.2588G>C
12	M	48	59	20/40	20/32	3A	3A	c.4670A>G; 6148G>C	c.3237T>C; 5603A>T
13	M	81	87	20/250	20/160	3B	3B	c.2549A>G; 4667+5G>T; 5882G>A^e^	c.5603A>T^e^
14	F	12	24	20/500	20/200	3C	3C	c.2626C>T	c.5714+5G>A
15^h^	F	56	66	20/125	20/200	3A	3A	c.517delC	c.587C>T^f^
16	M	46	74	20/32	20/40	2	3B	c.4139C>T^e^	c.5603A>T^e^
17	F	13	45	20/250	20/250	3B	3B	c.2966T>C^e^	c.67-1860A>G; c.6079C>T^e^
18	M	19	29	20/125	20/125	3B	3B	c.2588G>C; c.5603A>T	c.5461-10T>C; c.5603A>T
19^h^	F	20	59	20/400	20/230	3B	3B	c.3109G>T^e^	c.5761G>A^e^

^a^Individuals from the same family are indicated by a lower case letter following the same number; all were siblings except for subjects 3a and 3b, who were mother and son.

^b^BCVA; best-corrected visual acuity, as measured at imaging session.

^c^Modified Fishman grading scale: Grade 1: flecks limited to within 1DD of foveal centre with no atrophy, Grade 2: flecks beyond 1DD of fovea with no atrophy, Grade 3: Choriocapillaris atrophy of the macula associated with flecks within 1DD of foveal centre (3A), flecks beyond 1DD of fovea but within central 55° (3B) and flecks beyond 55° (3C), and Grade 4: Diffuse flecks and choriocapillaris atrophy throughout the fundus.

^d^No familial DNA for phase determination, four ABCA4 variants detected: c.5836-145C>A, c.4253+43G>A, c.5177C>A, c.5603A>T.

^e^Origin of parental alleles not established but familial analysis indicates variants are biallelic.

^f^Variant not detected in parent but familial analysis indicates variants are biallelic.

^g^Asymptomatic mother and son, age of onset is age at which retinal lesions were first identified.

^h^East Asian origin.

### Deep learning training and optimization procedure

**Figure 1:**
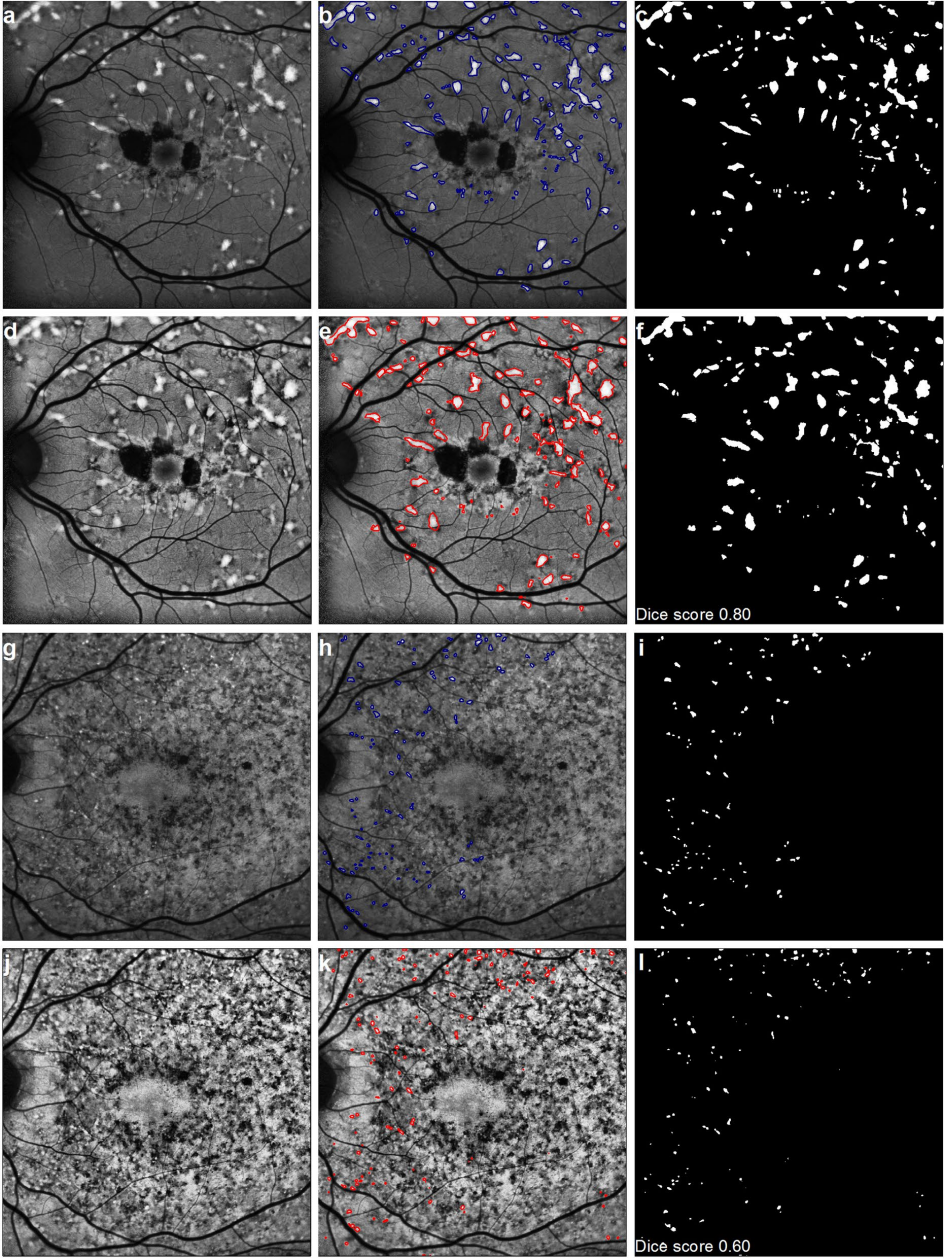
Manual and deep learning on Stargardt FAF images. (**a**) An example of a raw FAF image with discrete large pisciform lesions with (**b**) the outline of the hyperautofluorescent flecks manually marked. (**c**) An image mask of the fleck outline was generated for image analysis. (**d**) CLAHE transformation applied to the raw image in panel (**a**) followed by (**e**) fleck marking via deep learning. (**f**) Image mask of fleck outlines from deep learning was generated for image analysis. Dice score between the manual and deep learning segmentations is shown on the bottom left corner. (**g**–**i)** Manual and (**j**–**l**) deep learning segmentation of a FAF image with a diffusely speckled FAF pattern, as per panels (**a**–**f**), with dice score shown on the bottom left corner.

Amongst 47 FAF images, 31 had discrete well-defined pisciform lesions and 16 had diffusely speckled lesions without pisciform lesions (Table 2, Fig. 1). Manual segmentation of the flecks marked a total of 4833 lesions and the total lesion area was 88.2 mm^2^ in the 47 images. FAF images with a discrete, large pisciform classification had an average of 109 (range 10–249) fleck lesions with a mean total hyperautofluorescent area of 2.35 (range 0.29–5.55) mm^2^ while those with a diffusely speckled FAF classification had an average of 92 (range 5–202) lesions with a mean total area of 0.96 (range 0.05–2.04) mm^2^.

**Table 2:** Characteristics of FAF images used in deep learning training.

Subject ID^a^	Fleck lesion type	RE		LE		
		Fleck count	Fleck area (mm^2^)	Fleck count	Fleck area (mm^2^)	Dice score^b^
1^c^	Discrete	249	5.49	171	2.74	
1^c^	Discrete	n/a		152	2.02	
1^c^	Discrete	n/a		144	2.41	
2	Diffuse	128	0.70	149	1.39	
3a	Discrete	n/a		10	0.29	
3b	Discrete	167	4.85	207	4.85	0.77
4a	Discrete	53	0.69	59	0.79	0.72
4b	Discrete	159	4.36	233	3.71	
5	Discrete	78	2.59	57	2.44	
6	Discrete	202	2.11	249	2.76	
7a	Diffuse	202	2.04	182	1.34	
7b	Diffuse	109	1.38	147	1.29	0.61
8a	Diffuse	5	0.05	7	0.07	
8b	Diffuse	71	1.48	50	1.03	0.63
9a	Diffuse	37	1.03	109	1.57	
9b	Diffuse	47	0.35	102	0.68	0.60
10	Discrete	145	4.29	154	3.74	0.80
11	Discrete	101	2.02	124	2.17	0.70
12	Discrete	48	0.56	29	0.44	0.69
13	Discrete	34	0.73	35	0.79	0.57
14	Diffuse	57	0.43	67	0.48	0.33
15	Discrete	29	0.50	10	0.86	
16	Discrete	50	2.85	89	2.55	
17^c^	Discrete	n/a		64	0.71	
17^c^	Discrete	n/a		52	0.60	
18	Discrete	n/a		55	2.43	
19	Discrete	n/a		155	5.55	

*n/a* not applicable.

^a^Individuals from the same family are indicated by a lower case letter following the same number; all were siblings except for subjects 3a and 3b, who were mother and son.

^b^Dice score between manual and deep learning segmentation, only available in 10 left eye images used for validation.

^c^Longitudinal serial FAF images were used as part of the training set in subjects 1 and 17.

Of the 47 manually segmented FAF images, 37 were selected as the training and validation sets in deep learning and 10 used as independent testing images. The images were graded according to their clinical appearance and the senior author (FKC) ensured that, in the training, validation and testing sets, the distribution of clinical appearance was even across the three datasets. Fifty image patches (512 × 512 pixels) were generated from each of the 37 FAF images (total 1850 patches). Of these, 1750 and 100 partitioned image patches were used for training and validation, respectively. There was improvement in dice score and a decrease in validation loss with training. Both dice score and validation loss appeared to plateau from the 100th epoch onwards.

**Figure 2:**
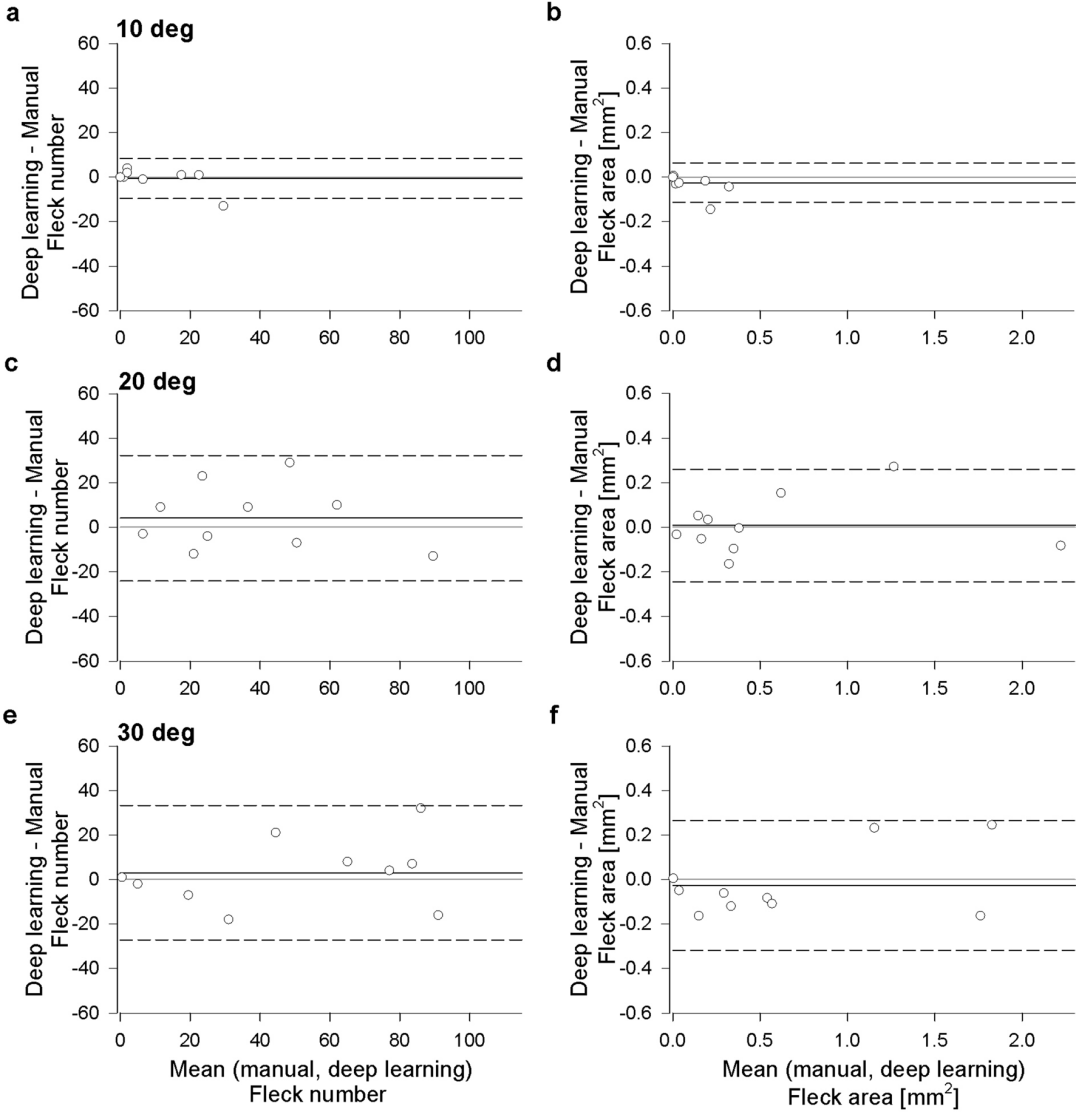
Bland–Altman plots comparing manual and deep learning segmentation methods. (**a**) Difference in fleck count (deep learning − manual) plotted against the mean of manual and deep learning fleck count in the central 10° ring. Solid black line indicates the mean difference and dashed black lines indicate the 95% confidence interval, gray line indicates no difference. (**b**) Difference in fleck area (deep learning − manual) plotted against the mean of manual and deep learning fleck area in the central 10° ring. Other details as per panel (**a**). (**c**,**d**) Bland–Altman plots of fleck number and fleck area in the 20° ring, respectively. Other details as per panel (**a**). (**e**,**f**) Bland–Altman plots of fleck number and fleck area in the 30° ring, respectively. Other details as per panel (**a**).

Bland–Altman analyses^16^ (Fig. 2) between manual and deep learning segmentation were performed in the 10 FAF images (4 with diffuse speckled and 6 with discrete fleck lesions) that underwent testing. Three concentric circles (10°, 20°, 30° diameter) centered at the fovea were placed on each image, which partitioned each image into a central disc of 10° diameter and two hollowed-out rings of 10°–20° and 20°–30° diameters. Manual and deep learning segmentation-derived fleck count were similar, with a mean difference (deep learning − manual) of − 0.60 (95% CI − 9.57 to 8.37), 4.10 (95% CI − 24.01 to 32.21) and 3.00 (95% CI − 27.21 to 33.21) for the 10° disc, 10°–20° and 20°–30° rings, respectively. Total fleck area was also similar, with a mean difference (deep learning − manual) of − 0.03 (95% CI − 0.11 to 0.06), 0.01 (95% CI − 0.24 to 0.26) and − 0.03 (95% CI − 0.32 to 0.26) mm^2^ for the 10° disc, 10°–20° and 20°–30° rings, respectively. The mean ± standard deviation (SD) dice score for FAF images with diffuse speckled patterns (N = 4) was lower than those with discrete flecks (N = 6); 0.54 ± 0.14 versus 0.71 ± 0.08, respectively (p < 0.04, *t* test).

### Longitudinal analysis

**Figure 3:**
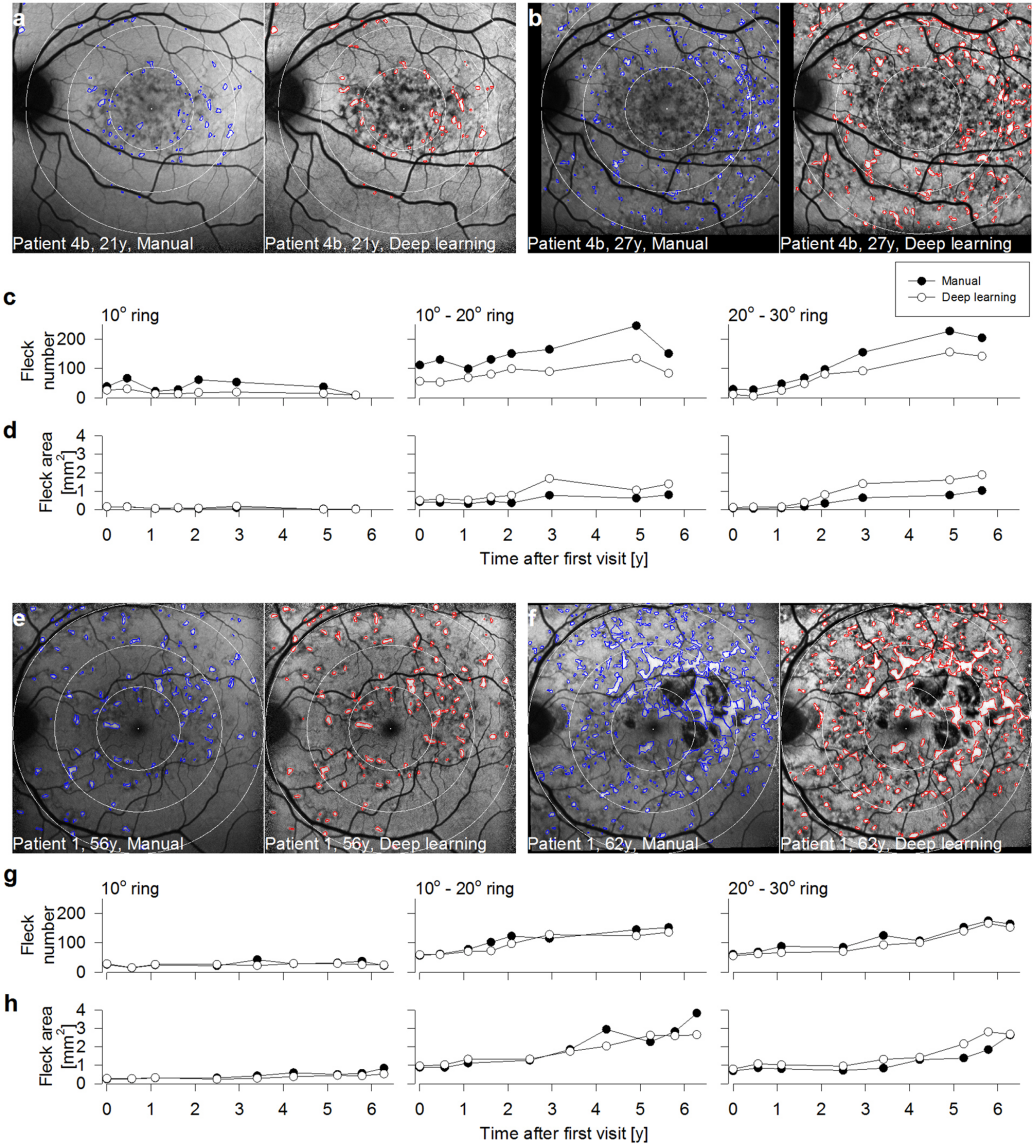
Two examples of longitudinal data analysed via manual or deep learning segmentation. (**a**) Manual (blue outlines) and deep learning (red outlines) segmentation of the hyperautofluorescent flecks of Patient 4b, 21 years old, at the first visit. Each image is sub-divided into three rings (10°, 10°–20°, 20°–30° diameter), centred on the fovea. (**b**) Manual and deep learning segmentation of the same eye as panel (**a**) 6 years later at 27 years old. All other details as per panel (**a**). (**c**) Fleck number plotted against time after first visit using manual (filled) and deep learning (unfilled) segmentation in the 10° (left), 10°–20° (middle) and 20°–30° (right) rings. (**d**) Fleck area plotted against time after first visit using manual (filled) and deep learning (unfilled) segmentation in the 10° (left), 10°–20° (middle) and 20°–30° (right) rings. (**e**–**h**) Manual versus deep learning longitudinal results in Patient 1, 56 years old at first visit and 62 years old at last visit. Other details as per (**a**–**d**).

Right and left eye images from 6 subjects with discrete pisciform flecks and a minimum of 12 months of follow-up (mean ± SD total follow-up period: 4.2 ± 1.8 years) underwent both manual and deep learning segmentation. Longitudinal analyses are illustrated using two case examples: Patients 4b and 1 (Fig. 3). Patient 4b at 21 years old showed discrete pisciform FAF regions at the first visit (Fig. 3a), which progressed into speckled hypoautofluorescent regions at the last visit (27 years, Fig. 3b). Manual (blue outlines) and deep learning (red outlines) segmentation results at each visit are shown side by side. In all images, three concentric circles (10°, 20°, 30° diameter) were centred on the fovea, from which the number and area of FAF flecks within each ring were analyzed. Fleck number (Fig. 3c) did not significantly increase over time in the 10° ring (p = 0.06), but the deep learning method underestimated the fleck count across most follow-up visits (p < 0.05). In the 20° and 30° rings, fleck number increased over time (both p < 0.05) and the deep learning method again underestimated the fleck count in both rings (both p < 0.05). Fleck area was low with either method in the 10° ring over the five years (Fig. 3d, left). In contrast, both methods detected an increase in fleck area over time (p < 0.05) in the 20° and 30° rings, with the deep learning method overestimating fleck area (both p < 0.05). Fleck progression was also shown in an older patient (Patient 1, 56 years at first visit) with discrete pisciform lesions regions at the first visit (Fig. 3e), which progressed into discrete regions of RPE atrophy centrally and speckled hypoautofluorescent regions in the perimacula at the last visit (Fig. 3f, 62 years). There was no increase in fleck number across time in the 10° ring (p = 0.15, Fig. 3g, left) and no difference in fleck number detected with either method (p = 0.47). In the 20 and 30° rings, both manual and deep learning methods showed a significant increase in fleck number over time (p < 0.05) but with no difference in fleck number estimation between the two methods (20° ring, p = 0.15; 30° ring, p = 0.07). Fleck area (Fig. 3h) was increased significantly over 6 years in all three rings (all p < 0.05). The 10° ring showed a significantly larger area with the manual method (p < 0.05), but there was no difference between deep learning and manual segmentation in the outer two rings (20° ring, p = 0.27; 30° ring, p = 0.06).

**Figure 4:**
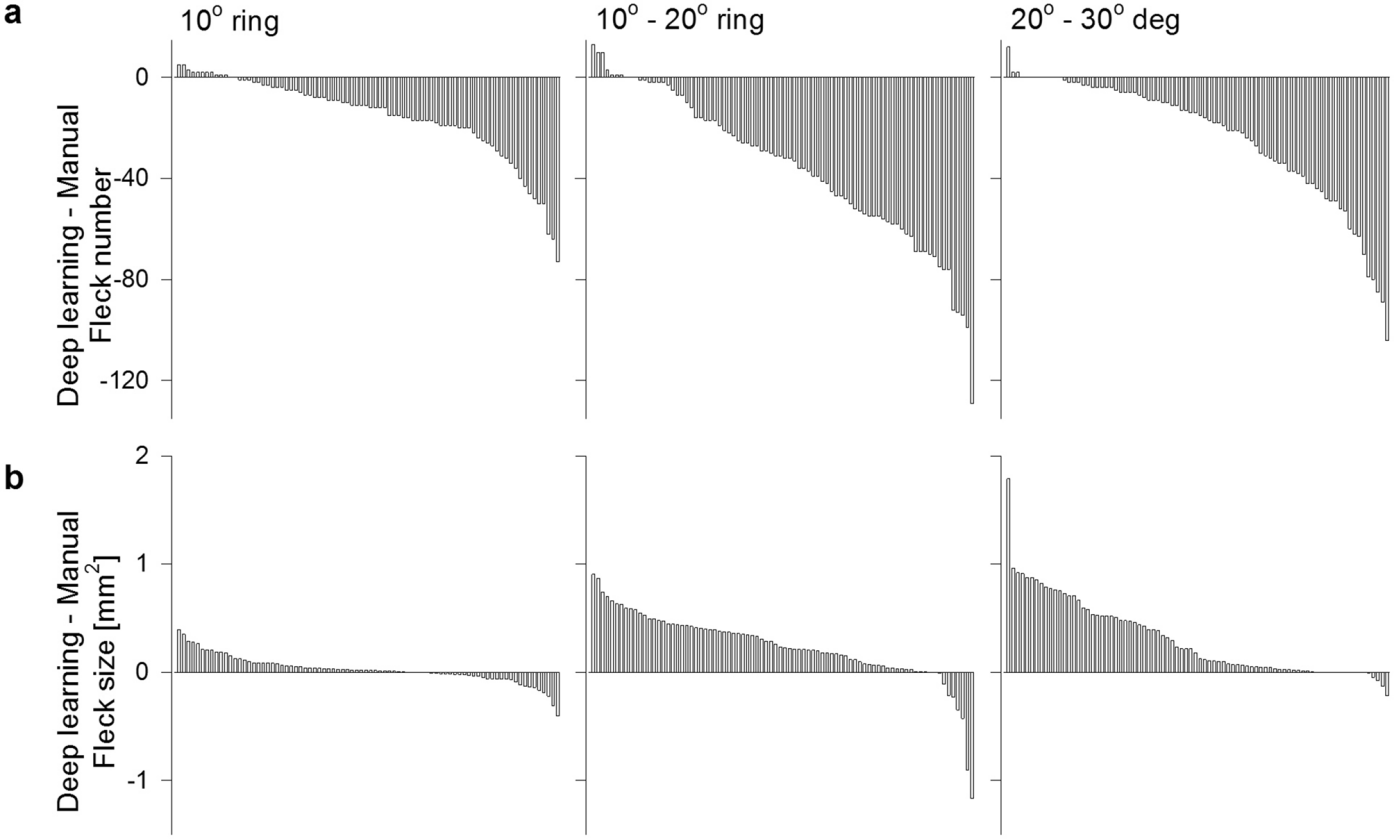
Manual versus deep learning image segmentation in longitudinal data. (**a**) Difference in fleck number between deep learning and manual segmentation in all 82 images available for longitudinal analysis within the 10° (left), 10°–20° (middle) and 20°–30° (right) rings. (**b**) Difference in fleck area between deep learning and manual segmentation in all 82 images available for longitudinal analysis, other details as per panel (**a**).

Overall, a total of 82 images across 12 eyes were used for longitudinal analysis. The deep learning segmentation method provided a lower fleck count than manual segmentation in 69 (84%), 72 (88%) and 70 (85%) of the 82 FAF images in the central 10° disc, 10°–20° and 20°–30° rings, respectively (Fig. 4). Conversely, the deep learning segmentation method provided a greater fleck area than manual segmentation in 52 (63%), 74 (90%) and 68 (83%) of the 82 FAF images in the central 10° disc, 10°–20° and 20°–30° rings, respectively (Fig. 4). The mean ± SD changes in fleck count derived from manual segmentation were − 1.5 ± 3.6, 8.9 ± 7.4, 13.4 ± 13.5 flecks/year for the 10° disc, 10°–20° and 20°–30° rings, respectively. Deep learning segmentation provided an estimated change in fleck count number ± SD by − 2.2 ± 3.4, 6.9 ± 3.3, 10.8 ± 9.8 flecks/year for the 10° disc, 10°–20° and 20°–30° rings, respectively. The mean ± SD change in fleck area derived from manual segmentation was − 0.01 ± 0.05, 0.05 ± 0.26, 0.12 ± 0.18 mm^2^/year for the 10° disc, 10°–20° and 20°–30° rings, respectively. Deep learning segmentation provided an estimated change in mean fleck area ± SD by − 0.02 ± 0.06, 0.07 ± 0.20 and 0.15 ± 0.25 mm^2^/year for the same three rings.

## Discussion

The U-Net deep learning architecture employed in the current study has been developed for biomedical image segmentation^17^, with various adaptations of the original architecture proposed, such as M-Net^18^, TernausNet (arXiv:1801.05746 [cs.CV]) and LinkNet (arXiv:1707.03718 [cs.CV]). To our knowledge, our study is the first to apply deep learning to quantify hyperautofluorescent fleck lesions in FAF images.

Several FAF parameters have been proposed for quantifying STGD1 severity or documenting natural history, including analysing the area of definitely or questionably decreased autofluorescence^9^, quantitative fundus autofluorescence^13^, fluorescence lifetime profile of FAF-detected flecks over time^19^ and expansion of the boundary of the hyperautofluorescent fleck^20^. The most popular of these currently is the growth rate of the central atrophic lesion^7,9,14,21,22^. In this work, we proposed an alternative method of disease severity assessment using quantitative analysis of hyperautofluorescent fleck lesions based on count and area. We purposely chose a wide variety of FAF images—those with well-defined or discrete pisciform lesions that were easy to manually segment as well as those with more diffuse and speckled FAF lesions that were much more difficult to identify—to include in the training and validation sets. Almost one third of the patients did not have any RPE atrophy, supporting the value of this method in assessing disease progression prior to atrophy formation. We observed that segmentation of flecks from images with diffusely speckled FAF regions tended to achieve poorer identification during validation, which was reflected in the dice scores shown in Table 2. This could be due to uncertainties in manual delineation and/or the lack of lesion feature similarity identified by the deep learning algorithm. Nevertheless, Bland–Altman analysis (Fig. 2) showed a good overall agreement between manual and deep learning in both fleck count and fleck area for the 10 FAF images used for testing, albeit with relatively wide 95% confidence intervals.

In longitudinal analysis with a larger number of consecutive FAF images, we observed that the deep learning algorithm tended to underestimate the number of fleck lesions but overestimate a greater fleck area compared to manual segmentation of the same image; this seems to contradict the Bland–Altman analysis based on the 10 test images. It is important to note that test images contain FAF images with diffusely speckled (N = 4) and discrete flecks (N = 6) whereas the longitudinal cohort only had images with the latter types of lesions, specifically chosen to illustrate the clinical utility of this method. We also observed that deep learning segmentation tended to ignore blood vessels that course over a large fleck and identify this as a single fleck lesion whilst manual segmentation delineated two separate lesions separated by the overlying blood vessel (Fig. 3a,b,e,f). We attribute this difference in fleck outline segmentation to the CLAHE transformation that was performed prior to segmentation using the deep learning method. The CLAHE transformation also tended to enhance the overall appearance of fleck lesions and this in turn obscured the shadow created by the overlying blood vessel, resulting in the identification of a single fleck lesion without blood vessel interference. One explanation for the increased area of fleck lesion using the deep learning method is that CLAHE enhances the appearance of lesions within the FAF image, which effectively enlarges the apparently hyperautofluorescent fleck lesion size. In addition, given that deep learning segmentation tended to incorporate the entire fleck lesion without interference from overlying blood vessels, the overall lesion area would have included the pixels representing blood vessels coursing over the fleck lesion.

To our knowledge, there are no data on the natural history of hyperautofluorescent fleck count and area in STGD1. The similar trends in the change in fleck count and area over time using both manual and deep learning segmentation suggests that deep learning algorithms may be useful in future studies of retinal fleck pathophysiology. The reduction in fleck count and area in the central 10° disc can be explained by the natural life cycle of flecks evolving into RPE atrophy^9,14,23^. In contrast, there was a general increase in fleck number and size over time in the outer rings as peripheral atrophy generally does not develop until later in the disease course^21,24^. Given that hyperautofluorescent flecks appear to increase gradually in number and size over time and precede the formation of central atrophy by several years in STGD1 course, further work towards validating fleck count and area as a potential clinical trials endpoint measure is warranted.

One major limitation of the study is the small number of FAF images used, due to the low number of these rare Stargardt patients. To overcome this drawback, we utilized a patch-based approach commonly used in deep learning methods to increase the training set sample size. However, our training set still had eyes with very few flecks and/or speckled FAF signals; future training sets should remove and replace these images with those that have the greatest number of well-defined pisciform fleck lesions. The second limitation is the use of CLAHE transformation in our deep learning workflow, which transformed the appearance of the hyperautofluorescent flecks when compared to the raw images used in manual segmentation. Future studies should either manually segment the FAF images after CLAHE or explore other image transformation techniques such as region erosion method that truthfully retain the appearance of the hyperautofluorescent flecks.

In conclusion, we have demonstrated that hyperautofluorescent flecks in FAF images may be a useful structural outcome measure in STGD1. More importantly, we trained and put forward a deep learning-based fleck segmentation method which is less time-consuming than manual marking. Further research to refine the deep learning algorithm for fleck segmentation is warranted given its potential as a clinical trials outcome measure in STGD1.

## Methods

The study adhered to the tenets of the Declaration of Helsinki and ethics approval was obtained from the Human Ethics Office of Research Enterprise, The University of Western Australia (RA/4/1/8932 and RA/4/1/7916) and the Human Research Ethics Committee, Sir Charles Gairdner Hospital (2001-053), Perth, Western Australia, Australia. Written informed consent was obtained from each individual for their imaging data to be used for research purpose.

### Patient selection

The Western Australian Retinal Degeneration (WARD) study database was interrogated for subjects with a molecular diagnosis of *ABCA4.* FAF images of these subjects were examined and suitable images were chosen for the study. Exclusion criteria included poor image quality and extensive atrophy resulting in no visible flecks within the macular 30° × 30° FAF image.

### Genetic analysis and pathogenicity assessment

Genomic DNA extracted from peripheral blood^25^ was analysed by targeted next-generation sequencing, using a Stargardt/Macular dystrophy panel (2014–2019 versions 1–5; 5–13 genes) or a retinal dystrophy (RD) NGS SmartPanel (version 4 or 7; 183 or 233 genes)^26^ targeting all exons and flanking intronic regions of known Stargardt/Macular dystrophy or retinal dystrophy genes, together with known *ABCA4* deep-intronic variants. Candidate variants were confirmed by Sanger sequencing. Sequencing was performed by Casey Eye Institute Molecular Diagnostics Laboratory (Portland, OR, USA) or Molecular Vision Laboratory (Hillsboro, OR, USA). Sequences were aligned to the *ABCA4* reference sequence NM_000350.2/3, with nucleotide 1 corresponding to the A of the start codon ATG, and described in accordance with Human Genome Variation Society recommendations version 15.11^27^. The phase of the variants was examined in the parents, where available, and family members. Variant pathogenicity was assessed as previously described^28^ and interpreted according to the American College of Medical Genetics and Genomics/Association for Molecular Pathology (ACMG/AMP) joint guidelines^29^.

### Fundus autofluorescence image acquisition

FAF images were acquired using a confocal scanning laser ophthalmoscope (Heidelberg Spectralis HRA without OCT, Heidelberg Engineering, Heidelberg, Germany) as previously described^30,31^. In brief, FAF (488 nm excitation, > 500 nm emission) images were acquired using a 30° × 30° acquisition window. During acquisition, the manufacturer’s averaging function (Automatic Real Time; ART), which averages multiple images in real time, was activated in order to maximize signal-to-noise ratio.

### Manual segmentation and analysis

Following image acquisition, raw images were extracted in bitmap format (1536 × 1536 pixels) and an ophthalmologist (FKC) marked the foveal centre and manually segmented individual autofluorescent flecks for each image using Paint.NET (v 4.2.9). Thirty-seven marked images served as training and validation sets for deep learning while 10 were used for testing. Prior to testing, parameters from each image (i.e. number of flecks, total area of flecks) were extracted. Fleck lesion size was calculated using the x- and y-axis scaling factor provided by the Spectralis software, which took into account the magnification factor of the eye due to differences in refractive power. This was done by creating an image mask of the fleck outline for each of the 10 FAF images, followed by placing three concentric circles (diameters of 10°, 20°, 30°) on each image, centred at the fovea. This allowed partitioning of all FAF images into three zones (10° disc, 10°–20° ring and 20°–30° ring), from which the two parameters extracted from each region served as reference data (i.e. ground truth).

Longitudinal analysis was performed in a subset of 12 eyes from 6 patients with discrete or well-defined pisciform flecks. These images were not used for deep learning training and testing. Following manual segmentation, image registration was first performed following manual segmentation of all available FAF images. A two-step image registration approach was utilized for registering a baseline image to its follow-up images. Given that the images acquired were macula-centred images with a large common area between them, at the first step, a simple rigid translation registration approach was applied to ensure that the baseline and each follow-up image align at the fovea. A 12-parameter transformation matrix was used to mimic the curved retinal surface via quadratic modelling. In the second registration step, a Dual-Bootstrap Iterative Closest Point (ICP) registration approach^32^ was used between the image pairs from the first step. The principle of Dual-Bootstrap ICP approach involves starting from an initial 12-parameter transformation estimate that is only accurate in a small region (i.e. bootstrap region). In each bootstrap region, the algorithm iteratively refines the transformation and expands this locally accurate initial alignment into a globally accurate alignment between the two images. Pixel-level registration accuracy was achieved by applying the two-step registration method. However, the limitations are that it was not suitable for eyes with large atrophic areas at baseline and eyes with large atrophic changes in their follow-up images. Flecks were quantified after registration by placing three concentric rings (radius 5°, 10° and 15°) centred at the fovea and measuring the number and the total area within each zone: 10° disc, 10°–20° ring and 20°–30° ring.

### Developing deep learning algorithm for fleck segmentation

**Figure 5:**
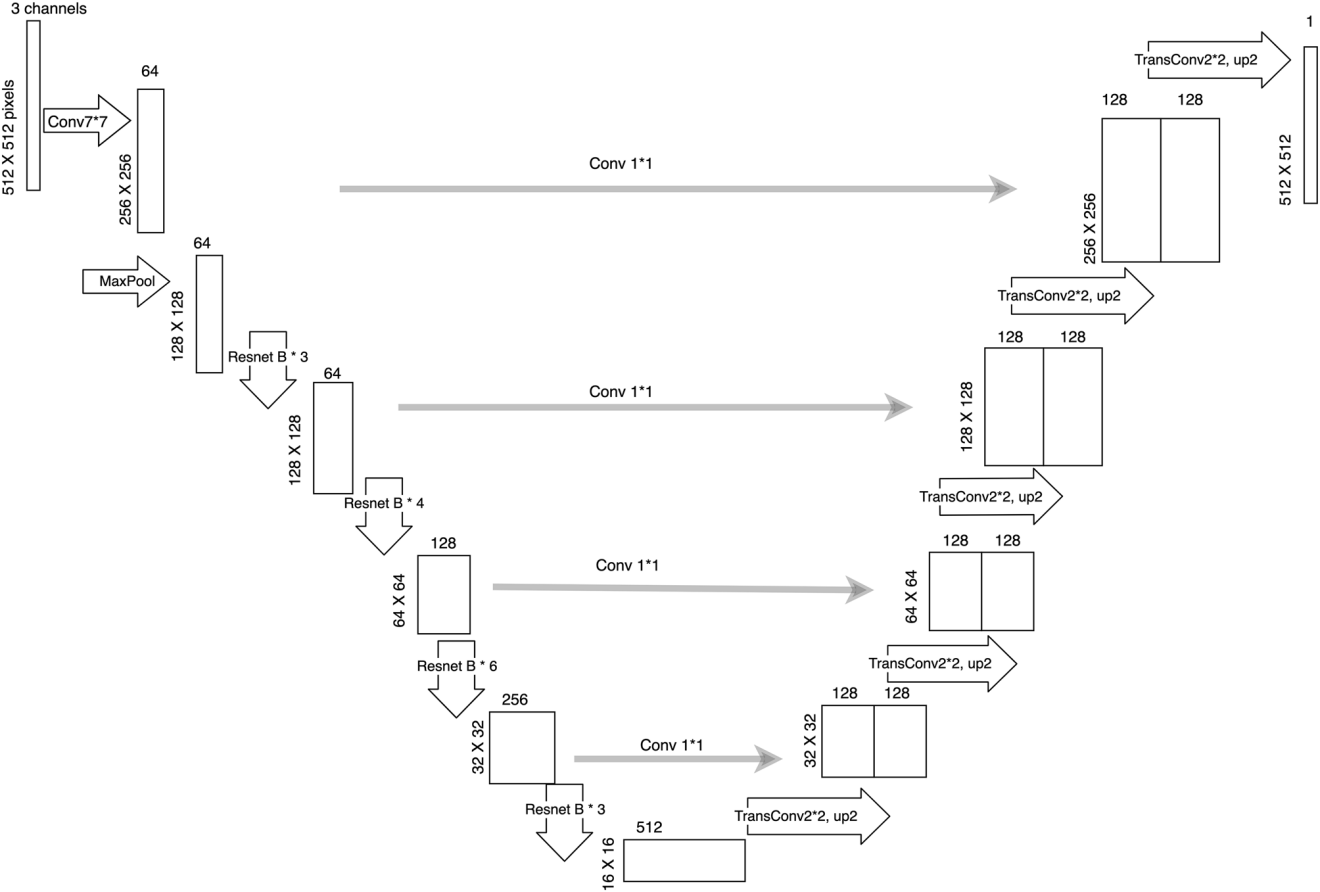
Architecture of ResNet-UNet. The structure of the ResNet-UNet uses the traditional UNet structure, which comprises of encoder (down-sampling) and decoder (up-sampling) portions. The down-sampling encoder is replaced by the ResNet-34 model. Conv n*n indicates n × n convolutional layer. TransConv 2*2, up 2 indicates 2 × 2 transposed convolution and keeping stride of 2.

A UNet model with ResNet encoder (ResNet-UNet) was constructed for the deep learning fleck segmentation. The architecture of the ResNet-UNet follows traditional UNet's structure^17^, composing of encoder (down-sampling) and decoder (up-sampling) portions. The key difference between the ResNet-UNet and the UNet is that the down-sampling encoder adopts the ResNet-34 model^33^, which is widely applied in image classification area and benefits from the advanced deep residual learning method. The ResNet-34 used here consists sequentially of firstly a 7 × 7 convolutional layer (CL), a max pooling layer, and the following 16 residual blocks. Each residual block contains two 3 × 3 convolutional layers with ReLu and a batch normalization identity shortcut connection (Fig. 5). Therefore, the ResNet down-sampling portion totally consists of 34 layers. To match the image size changes [32, 64, 128, 256] in the up-sampling portion, the corresponding 4 feature maps from the down-sampling structure are: (1) output from the 7 × 7 CL (feature map size 256 × 256); (2) output from the first 3 residual blocks (feature map size 128 × 128); (3) output from the second 6 residual blocks (feature map size 64 × 64); (4) output from the third 4 residual blocks (feature map size 32 × 32). The up-sampling portion starts from the 512-channel 16 × 16 feature map. It is convoluted by a 2 × 2 transposed convolution with up-sampling factor 2 (stride = 2). The up-sampled output (128-channel 32 × 32 feature map) is concatenated with 1 × 1 convolutional output (128-channel) of the corresponding feature map from the down-sampling counterpart. The procedure is repeated until the output reaches image size 256 × 256. Then, the final layer transposes the 256-channel 256 × 256 feature map to a 1-channel 512 × 512 feature map, giving a probability map of the segmented objects.

Contrast Limited Adaptive Histogram Equalization (CLAHE)^34^ was applied to the raw FAF images prior to deep learning training. The number of images in the training set was increased by extracting image patches from cropped portions (512 × 512 pixels) of the full-size CLAHE-FAF images. The training set was further augmented by using patches that have been randomly (1) rotated through 90°, 180° and 270°; (2) inverted horizontally or vertically; and (3) magnified by a scale of 1.0–1.3. The overall model was trained in two stages in order to take advantage of the ResNet-34^33^ with its pre-trained weights. The first stage involved freezing the down-sampling encoder portion of the model and only optimized the weights of the up-sampling decoder portion. For the second stage, the ResNet-34 down-sampling portion was unfrozen to fine-tune the weights of the entire model. The learning rates were adjusted during the training. Adam optimization^35^ and BCEWithLogitsLoss were adopted in the overall training process. Dice score between the learning results and the ground truth was used as performance metric. The model was trained on Google cloud (Google Colab). The computing configuration was a single 12 GB NVIDIA Tesla K80 GPU. The training data batch size was set as 6. The initial training rate was 0.001 and the learning rate was adjusted during the training for the different layers. The model training was completed within 11 h (138 epochs).

### Applying deep learning algorithm to segment and analyze hyperautofluorescent flecks

The FAF images were first processed by CLAHE. The size of the original images was 1536 × 1536 pixels, which was partitioned into 9 non-overlapping image patches (3 × 3 grid, each grid 512 × 512 pixels). Each 512 × 512 pixel image patch was rotated 90°, 180° and 270° to generate 3 new image patches. This was followed by horizontally flipping the original image patch then rotation of the flipped image by 90°, 180°, 270° to generate 4 more image patches. All 8 patches were processed by the ResNet-UNet deep learning model for hyperautofluorescent region segmentations and consequently 8 outputs were obtained. The final lesion mask for the patch is the average of the 8 patches from the reverse transformations of the 8 model outputs. The hyperautofluorescent mask of the entire image was then formed from the 9 partitioned 512 × 512 pixel image patches.

**Figure 6:**
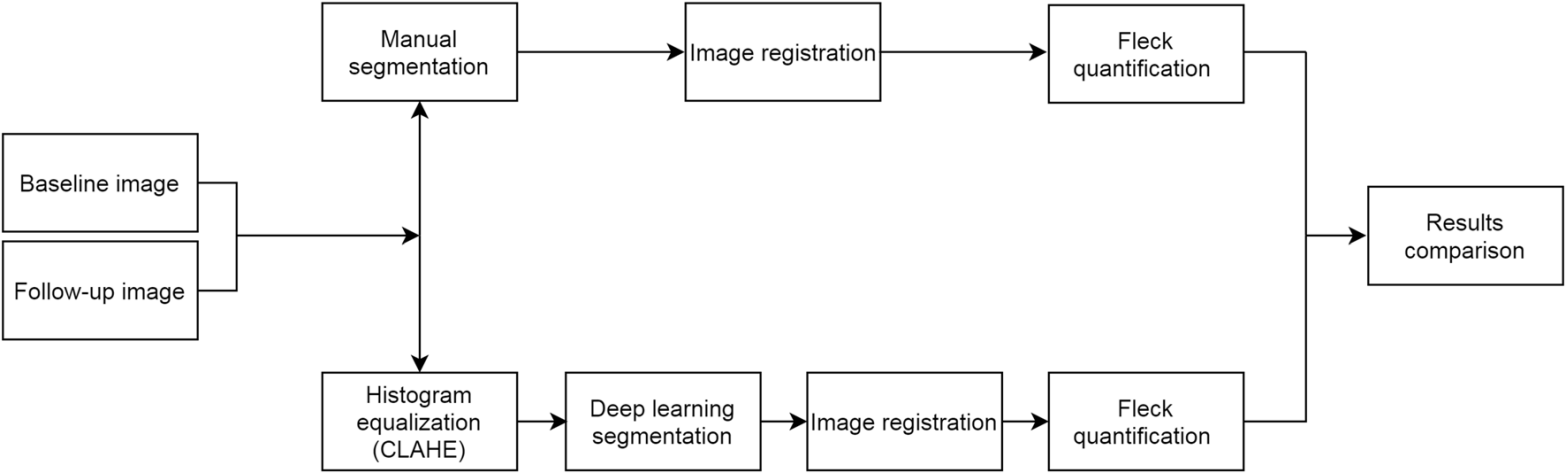
Workflow of longitudinal image processing for hyperautofluorescent flecks for both manual and deep learning segmentation. Baseline and follow-up images were segmented via both manual and deep learning before image registration. Fleck quantifications from both methods were then compared against each other.

Longitudinal analysis was performed using deep learning in the same subset of 12 eyes with manually segmented flecks. Following the delineation of the fleck outlines by deep learning, a mask of the fleck outline was generated and the same registration approach described in the manual segmentation section was utilized to measure the number and the total size of flecks within the three concentric rings (radius 5°, 10° and 15°) centred at fovea. Figure 6 illustrates the workflow for both manual and deep learning segmentation employed in the study.

### Validation outcome measure

In order to evaluate the model’s segmentation performance of the lesions, dice score (i.e. Intersection over Union, Eq. 1) was used, where the dice score (*D*) is equal to two times the common elements between the manual (*Y*) and deep learning (*Ŷ*) results divided by the total number of elements in each set.

1 $$D(Y,\;\hat{Y}) = \frac{{2|Y \cap \hat{Y}|}}{{|Y| + |\hat{Y}|}}.$$

Dice score was calculated for each image by comparing manually delineated regions to that marked by deep learning.

### Statistical analysis

Bland–Altman analysis^16^ was employed to evaluate the difference in parameters extracted from deep learning and manual segmentation methods in the testing set. In a subset of 12 eyes from 6 subjects, disease progression over time was assessed by using both deep learning and manual segmentation methods. Fleck count and area were extracted from all images from both methods and were compared across time via two-way ANOVA. All data were summarized as mean ± SD.

## Data Availability

The data that support the findings of this study are from the WARD study and restrictions apply to the availability of these data so are not publicly available. Data are however available from the authors upon reasonable request and with permission of the WARD study.
